# Activity of CdTe Quantum-Dot-Tagged Superoxide Dismutase and Its Analysis in Capillary Electrophoresis

**DOI:** 10.3390/ijms22116156

**Published:** 2021-06-07

**Authors:** Natalia Zaręba, Łukasz Lewandowski, Dominika Kunachowicz, Rene Kizek, Marta Kepinska

**Affiliations:** 1 Department of Biomedical and Environmental Analysis, Faculty of Pharmacy, Wroclaw Medical University, Borowska 211, 50-556 Wrocław, Poland; natalia.zareba@umed.wroc.pl (N.Z.); lukasz.lewandowski@umed.wroc.pl (Ł.L.); kizek@sci.muni.cz (R.K.); 2 Students Scientific Association, Department of Biomedical and Environmental Analysis, Faculty of Pharmacy, Wroclaw Medical University, Borowska 211, 50-556 Wrocław, Poland; d.kunachowicz@gmail.com; 3 Department of Human Pharmacology and Toxicology, Faculty of Pharmacy, Masaryk University, Palackeho 1946/1, 612 42 Brno, Czech Republic

**Keywords:** quantum dots, nanoparticles, superoxide dismutase, protein labeling, capillary electrophoresis, enzyme activity

## Abstract

Quantum dots (QDs) have a broad range of applications in cell biolabeling, cancer treatment, metastasis imaging, and therapeutic drug monitoring. Despite their wide use, relatively little is known about their influence on other molecules. Interactions between QDs and proteins can influence the properties of both nanoparticles and proteins. The effect of mercaptosuccinic acid-capped CdTe QDs on intercellular copper–zinc superoxide dismutase (SOD1)—one of the main enzymatic antioxidants—was investigated. Incubation of SOD1 with QDs caused an increase in SOD1 activity, unlike in the case of CdCl_2_, which inhibited SOD1. Moreover, this effect on SOD1 increased with the size and potential of QDs, although the effect became clearly visible in higher concentrations of QDs. The intensity of QD-SOD1 fluorescence, analyzed with the use of capillary electrophoresis with laser-induced fluorescence detection, was dependent on SOD1 concentration. In the case of green QDs, the fluorescence signal decreased with increasing SOD1 concentration. In contrast, the signal strength for Y-QD complexes was not dependent on SOD1 dilutions. The migration time of QDs and their complexes with SOD1 varied depending on the type of QD used. The migration time of G-QD complexes with SOD1 differed slightly. However, in the case of Y-QD complexes with SOD1, the differences in the migration time were not dependent on SOD concentration. This research shows that QDs interact with SOD1 and the influence of QDs on SOD activity is size-dependent. With this knowledge, one might be able to control the activation/inhibition of specific enzymes, such as SOD1.

## 1. Introduction

Quantum dots (QDs) are semiconductor nanocrystals, often composed of atoms of elements from groups II–VI, or III–V [1]. They are characterized by physical dimensions smaller than the exciton Bohr radius [2,3], thus the values of their confinement energy of the hole and the excited electron are related to their size [3,4]. Therefore, as the size of QDs increases, their absorption onset and fluorescence spectrum shift from blue to red [5]. Due to the ability to influence the duration and temperature of the synthesis and the type of ligands used for the synthesis, the size and shape of QDs can be controlled [6]. QDs have a high extinction coefficient [5], broad absorption spectra, high photostability, and tight and symmetrical photoluminescence spectra as against organic fluorophores [7], as well as a sharper density of states than higher-dimensional structures [8]. Moreover, QDs can be modified (e.g., by adding layers to their surface or by bioconjugation of ligands), which affects their physicochemical properties, such as water solubility and biocompatibility [9]. Uncoated QDs are more reactive and may react randomly with biomolecules [10]. Due to their characteristics, QDs have many applications, among which are biolabeling and bioimaging of molecules [11,12,13,14] or specific types of cells and tissues [15,16,17,18,19,20,21,22], therapeutic drug monitoring [23,24,25,26], assaying for copper [27,28] and various compounds, including picric acid [29], glucose [30], phenol, and hydrogen peroxide [31]. Moreover, the latest research aims to use QDs as drug carriers [26,32,33]. The fact that QDs have many applications, both in vivo and in vitro, creates a need for determining the influence of these nanoparticles on biomolecules, cells, and tissues. In addition to their cadmium release and direct induction of oxidative stress, CdTe QDs, similarly to cadmium salts and several environmental xenobiotics [34], have lately been shown to have an influence on one of the main enzymatic antioxidants—copper–zinc superoxide dismutase (Cu,Zn-SOD) [35]. In humans, Cu,Zn-SOD occurs in two forms (isoenzymes): intercellular (dimeric)—SOD1, found mainly in the liver, kidneys, heart, adrenal glands, and erythrocytes, and extracellular (tetrameric)—SOD3, found mainly in the lungs, colon, adipose tissue, lymph nodes, and plasma. Their role is to process superoxide radicals into oxygen and hydrogen peroxide, thus preventing an excessive concentration of reactive oxygen species (ROS) [36]. The relationship of the balance disturbances between the processes of reduction and oxidation with a deficiency in SOD in various diseases is the reason why many researchers became interested in this enzyme [37,38]. Catalytic antioxidants could help with the treatment of these diseases by protecting normal tissues without compromising treatment efficacy. The use of SOD mimetics to remove excess ROS is considered in the context of diseases such as atherosclerosis, diabetes, and neurodegenerative diseases and as an adjunctive therapy in transplantation, radiotherapy, and chemotherapy [39].

To our knowledge, there is no research into the connection between the size or potential of CdTe QDs and how they affect Cu,Zn-SOD activity or into assessing SOD1 labeling with CdTe QDs analyzed using capillary electrophoresis (CE) with laser-induced fluorescence (LIF) detection. This research aims to find out whether MSA-capped CdTe QDs of different sizes and potentials would have an influence on the activity of SOD1, compared to SOD1 inhibition by cadmium (II) chloride (CdCl_2_), and to analyze the interaction of SOD1 with CdTe QDs.

## 2. Results

### 2.1. Physicochemical Characteristics of CdTe QDs

**Figure 1:**
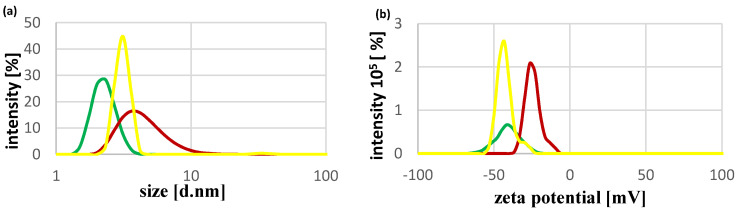
Size (**a**) and zeta potential (**b**) of green, yellow, and red MSA-capped CdTe QDs. The colors in the chart correspond to the colors of the QDs. (**a**) Logarithmic scale is used on the x-axis.

**Figure 2:**
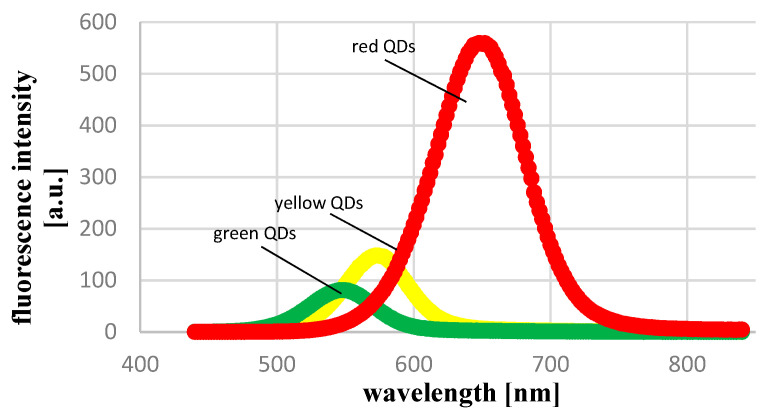
Fluorescence spectra of MSA-capped CdTe QDs.

The sizes of green, yellow, and red MSA-capped QDs, determined with the use of dynamic light scattering, were: 2.3 nm, 3.1 nm, and 4.6 nm, respectively, and their potentials were: −40.8 mV, −42.8 mV, and −24.4 mV, respectively. These data are shown in Figure 1. With the increasing size of QDs, a shift in their fluorescence spectra, from green (550 nm) to red (650 nm), could be seen (Figure 2).

### 2.2. The Activity of SOD1 in the Presence of CdCl_2_ or CdTe QDs

**Figure 3:**
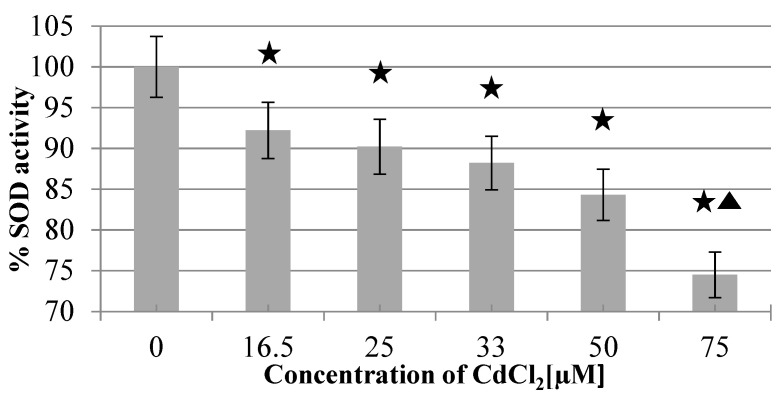
Inhibition of Cu,Zn-SOD activity upon 30 min of preincubation with aqueous CdCl_2_ solution at 30 °C. The asterisk marks statistically significant differences compared with the SOD activity without CdCl_2_. The triangle marks a statistically significant difference compared with the SOD activity in the presence of 16.5, 25, and 33 µM CdCl_2._ (*p* < 0.05).

There was no significant interference of CdCl_2_ (up to 33 µM) with the assay. However, 50 µM and 75 µM concentrations of CdCl_2_ caused interference by increasing the rate of adrenochrome formation by 2.75% and 11.9%, respectively. SOD1 (40 ng/mL) inhibited the process of adrenochrome formation by 49%. This inhibition was reversed upon exposing SOD1 to increasing concentrations of CdCl_2_. The mean % of SOD1 (40 ng/mL) activity inhibition by CdCl_2_ in concentrations of: 16.5 µM, 25 µM, 33 µM, 50 µM, and 75 µM was: 7.8%, 9.8%, 11.8%, 15.7%, and 25.5%, respectively, as shown in Figure 3.

**Figure 4:**
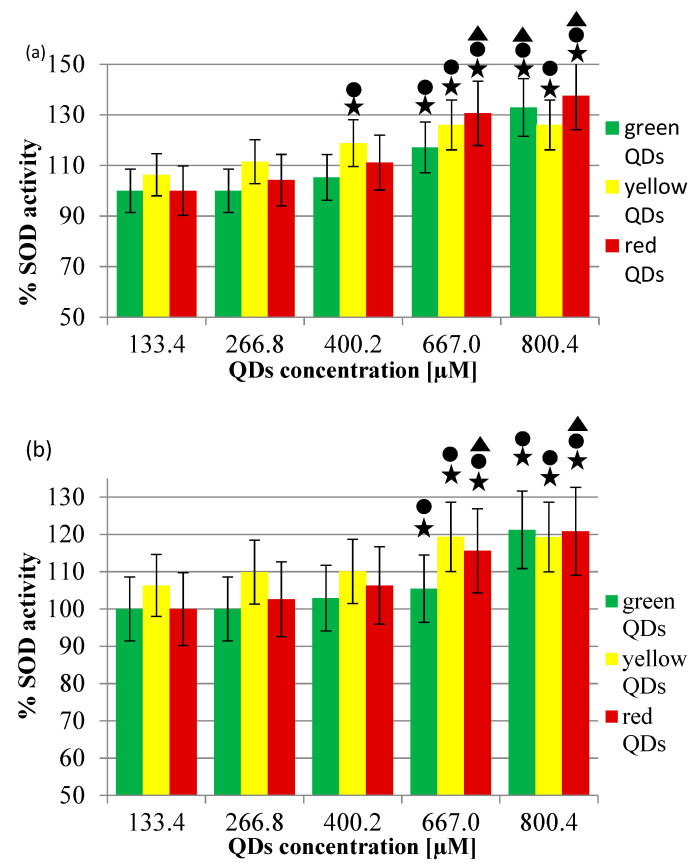
Changes in SOD activity, in the presence of MSA-capped CdTe QDs, after 30 min of preincubation at 30 °C: (**a**) raw data; (**b**) data after correction by % of QD interference. The activity of SOD without QDs accounted for 100%. The asterisk marks a statistically significant difference compared with the activity of SOD in the presence of a 133.4 µM QD concentration (*p* < 0.05)_._ The circle marks statistically significant differences compared with the activity of SOD complexes in the presence of a 266.8 µM QD concentration (*p* < 0.05). The triangle marks statistically significant differences compared with the activity of complexes in the presence of a 400.2 µM QD concentration (*p* < 0.05).

Upon addition of CdTe QDs to the reaction mixture, the rate of epinephrine autooxidation decreased. No changes in autooxidation rate were noticed after preincubation of SOD1 (40 ng/mL) with QDs in a concentration of 60.03 µM or 73.37 µM. However, preincubation of SOD1 with a higher concentration of QDs decreased the autooxidation rate. This effect varied, depending on the concentration and type (thus size and potential) of QDs, as shown in Figure 4. Generally, the higher the concentration of any type of the used QDs, the more a decrease in the epinephrine autooxidation rate was noticed. A greater decrease in the epinephrine autooxidation rate is associated with a larger diameter and a higher zeta potential of QDs (Figure 4). However, this effect is clearly visible in higher concentrations of QDs (667.0 µM, 800.4 µM).

### 2.3. Analysis of the Interaction of SOD1 with QDs with the Use of Capillary Electrophoresis

**Figure 5:**
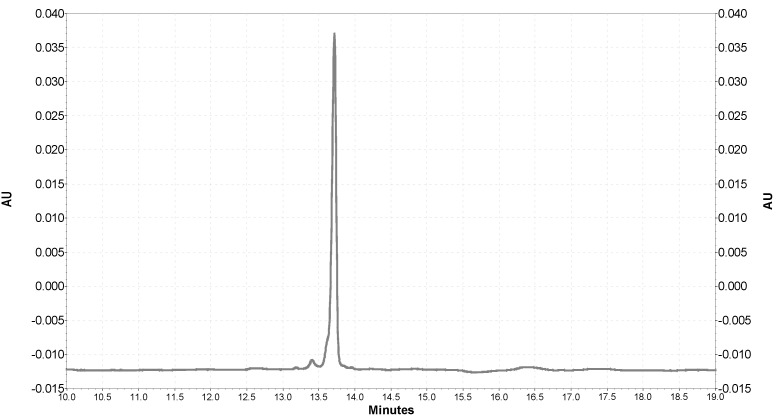
An electrophoretic diagram of SOD1 separation using CE with a UV detector (200 nm).

In the SOD1 separation analysis using CE with UV detection, the migration time was 13.8 min (Figure 5). 

**Figure 6:**
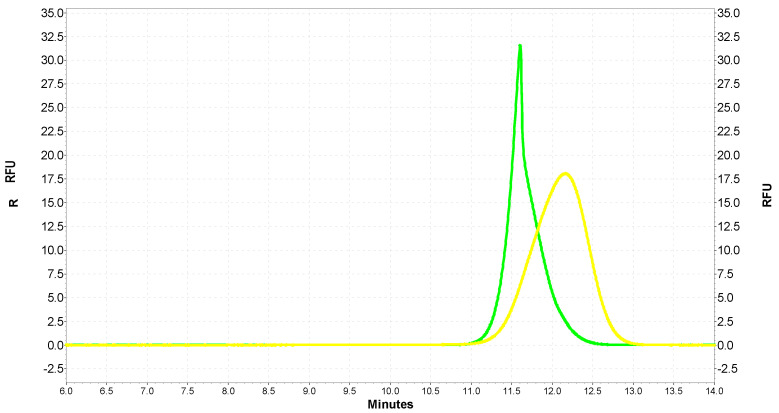
An electrophoretic diagram of G-QD (green) and Y-QD (yellow) separation using CE with an LIF detector.

Two types of QDs with similar dimensions and zeta potentials (green: G-QDs—2.3 nm, −40.8 mV; yellow: Y-QDs – 3.1 nm, −42.8 mV) and four different concentrations of SOD1 (1.2 µg/mL; 4 µg/mL; 40 µg/mL; 400 µg/mL) were selected for the analysis. The separation results of Y-QDs and G-QDs were: 21 RFU in 12.1 min and 40 RFU in 11.6 min, respectively (Figure 6).

**Figure 7:**
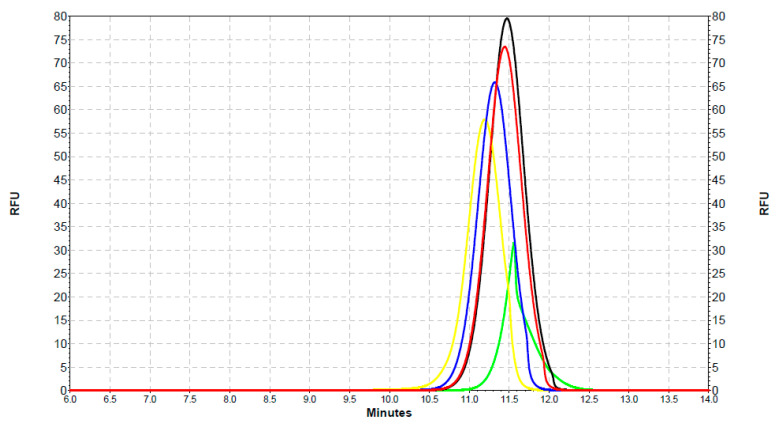
An electrophoretic diagram of separation of G-QDs–SOD complexes using CE with an LIF detector. Color codes: black: G-QDs with SOD (1.2 µg/mL), red: G-QDs with SOD (4 µg/mL), blue: G-QDs with SOD (40 µg/ml), yellow: G-QDs with SOD (400 µg/mL), and light green: G-QDs alone.

G-QDs with SOD1 separation showed the highest value of fluorescence when the lowest concentration of SOD was applied, and the lowest value of fluorescence for G-QDs alone (Figure 7).

**Figure 8:**
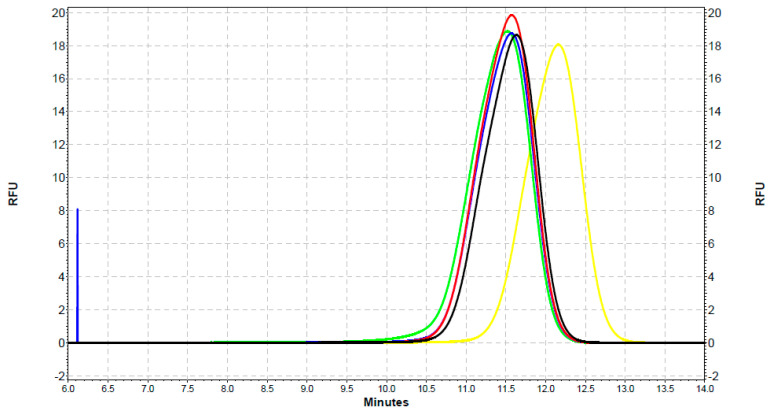
An electrophoretic diagram of the separation of Y-QDs–SOD complexes using CE with an LIF detector. Color codes: black: Y-QDs with SOD (1.2 µg/mL), red: Y-QDs with SOD (4 µg/mL), blue: Y-QDs with SOD (40 µg/mL), green: Y-QDs with SOD (400 µg/mL), and yellow: Y-QDs alone.

For the samples of Y-QDs with SOD1, the lowest fluorescence intensity occurred for Y-QDs alone as in the case of the G-QDs analysis and was equal to 18.1 RFU in 12 min. However, the highest measured signal intensity value was 19.9 RFU in 11.57 min for the 4 µg/mL concentration of SOD1 (Figure 8). 

**Figure 9:**
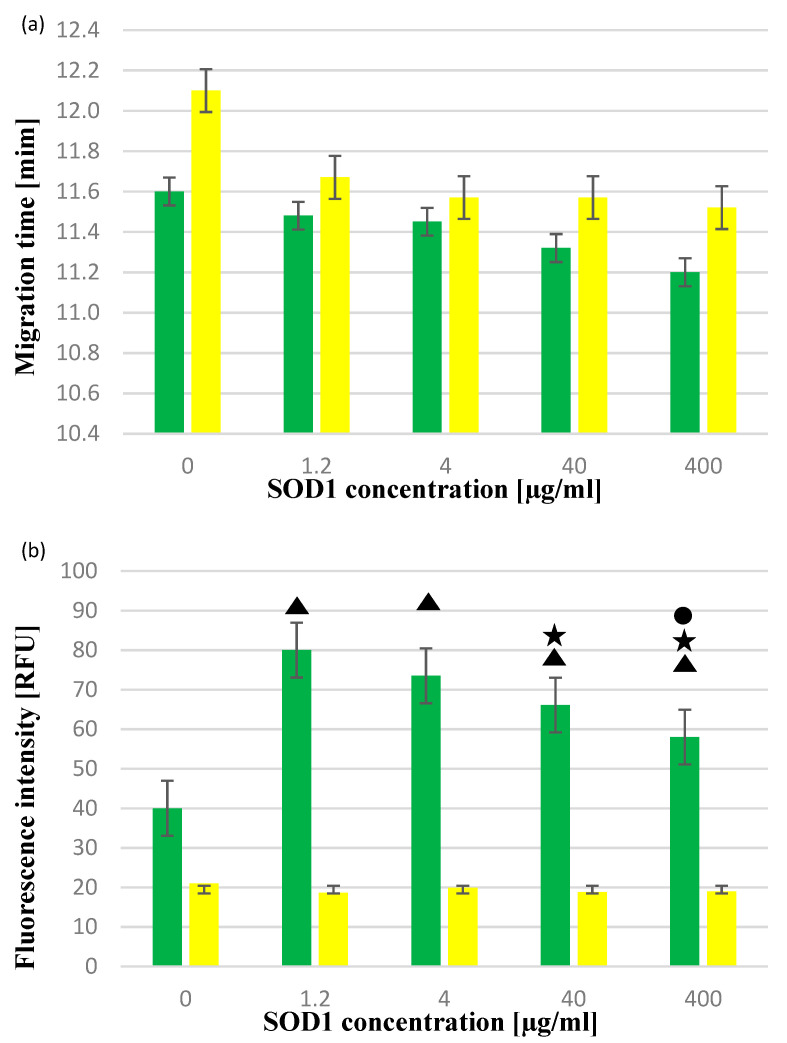
Dependence of (**a**) migration time and (**b**) fluorescence intensity signal on SOD1 concentration. The colors in the chart correspond to the colors of QDs. The triangle marks a statistically significant difference compared with the fluorescence of G-QDs alone (*p* < 0.05)_._ The asterisk marks statistically significant differences compared with the fluorescence of complexes in the presence of a 1.2 µM SOD concentration (*p* < 0.05). The circle marks a statistically significant difference compared with the fluorescence of complexes in the presence of a 4 µM SOD concentration (*p* < 0.05).

The migration time of SOD1–QDs complexes for all SOD1 concentrations was shorter compared with the migration time of the corresponding QDs alone (Figure 9). Comparing the electrophoretic mobility of all SOD1–Y-QDs and SOD1–G-QDs complexes, it was noticed that G-QD labeling caused signal shifts in the separation time for each of the SOD1 concentrations, and the higher the SOD1 concentration, the shorter the separation time, while the separation of SOD1–Y-QDs for all SOD1 concentrations was more or less equal. There was no statistically significant difference between migration times of complexes (Figure 9a). Regardless of the concentration of SOD1 used, statistically significant differences were observed in the fluorescence intensity of all SOD–YQDs complexes compared with SOD–GQDs (Figure 9b).

## 3. Discussion

Samia et al. have pointed out that CdSe QDs can generate reactive singlet oxygen species, and thus could be used in the future as photodynamic sensitizers in cancer therapy [40]. Thorough research by Ipe et al. showed that CdSe QDs, upon irradiation, produced less free radicals than CdS QDs [41]. Interestingly, CdSe/ZnS core/shell QDs did not produce more than ≈50 nM (sensitivity of the used assay) [41]. Nanoparticles in general may affect the structure of proteins, interacting with their C=O, C-N, and N-H groups, as shown on human serum albumin [42,43]. The interaction between QDs and human serum albumin may be connected to adsorption and complex formation in the case of negatively charged QDs. However, in the case of positively charged QDs, this interaction leads to the aggregation of QDs and, thus, adsorption of larger amounts of protein, causing fluorescence quenching and structural rearrangement of the protein [42,43]. Since the total oxidative capacity is maintained by proper activity of various enzymes, QDs may presumably induce oxidative stress by affecting such proteins, decreasing their activity. In the case of cadmium-containing QDs, one of the sources of oxidative stress may be Cd itself, as it may be released from the structure of QDs. This thesis seems plausible, as cadmium salts (mainly CdCl_2_, Cd(NO_3_)_2_) have been shown to inhibit three main enzymatic antioxidants: catalase, glutathione peroxidase, and superoxide dismutase [44,45,46,47,48,49,50]. Nevertheless, the release of Cd from QDs is induced by the oxidation of the QD core—it would be impossible to determine how much Cd would be released in the case of in vivo exposure to QDs, as the antioxidative capacity varies between individuals [51].

In the first part of this study, MSA-capped CdTe QDs were used to determine whether the used QDs would alter the activity of bovine SOD1. Dynamic light scattering (DLS) was used to determine the size (diameter) of QDs: 2.3 nm, 3.1 nm, and 4.6 nm in the case of green, yellow, and red QDs, respectively. The size of our QDs slightly differs from those found in other studies. In research carried out by Wang et al., the size of MSA-capped CdTe QDs of a fluorescence spectrum of 657 nm (red QDs) was approximately 3.9 nm [52]. A similar size (3.0–4.0 nm) of CdTe/MSA QDs was determined by Yu et al. using high-resolution transmission electron microscopy (HRTEM) [53]. However, in a study by Dutta et al., the size of MSA-capped CdTe QDs also measured using HRTEM ranged from approximately 5.0 nm to 6.0 nm [29]. According to Guszpit et al., the size of green, yellow, and red MSA-capped CdTe QDs, measured by dynamic light scattering, was: 3.8 nm, 4.5 nm, and 5.2 nm, respectively [54]. In the case of both studies, transmission electron microscopy or HRTEM was used to determine the size of QDs. The difference between the aforementioned results and the results of our research may be due to the different synthesis or/and analysis methods used.

The inhibitory action of CdCl_2_ on SOD1 has been shown to be dose-dependent. The lowest concentration of CdCl_2_, which inhibited SOD1, was 16.5 µM (inhibition by 6.40%), whereas other research, by Huang et al., showed inhibition of SOD1 in 30 nM of CdCl_2_ [48]. Such a discrepancy between the observed results may be due to the different SOD activity assay methods used, and different SOD origins (bovine SOD1 vs. human SOD1). Interestingly, another cadmium salt, Cd(NO_3_)_2_, was proven to increase SOD activity in several in vivo research studies [55,56]. The previously mentioned results obtained by Huang et al. indicate that the amount of Cd released from Cd-containing QDs in vivo may be sufficient to inhibit SOD1; QDs may be phagocytized by monocytes/macrophages, and thus exposed to highly oxidative environments [48]. According to research by Derfus et al., 24 h of incubation of CdSe QDs (0.25 mg/mL) with 1 M of H_2_O_2_ caused the release of 24 ppm of Cd [57], which is approximately 0.655 µM (CdCl_2_)—a concentration that could inhibit SOD1 [48].

Bovine SOD1 was incubated with three different types of MSA-capped CdTe QDs: green, yellow, and red (listed from the smallest to the largest in diameter). The results obtained suggest that the used CdTe QDs activate SOD1 in vitro. Presumably, this effect may depend on the size of QDs; the larger the size of QDs, the higher the activation of SOD (as seen in Figure 4). We checked whether QDs would interfere with the SOD assay method used and found that QDs interfered with the assay, decreasing the rate of the autooxidation of epinephrine. Not taking this fact into account could result in false assumptions, as the interference of QDs with the assay method could be understood as the activation of SOD (Figure 4). There have been several research studies assaying for SOD activity in the presence of Cd-containing QDs, although each of them features QDs with a different structure. Lu et al. [58] tested the antimicrobial activity of MSA-capped CdTe QDs—in this context SOD activity was measured ex vivo as one of the parameters needed for the survival of *Escherichia coli*—by preventing oxidative damage to the bacterial cell membrane. SOD activity was negatively affected by the exposure to QDs in a concentration of 120 nM and higher (160 nM, 200 nM); there was a significant decrease in the expression of SOD A/16S rRNA after exposure of bacteria to 200 nM of QDs. Interestingly, a slight increase in SOD activity was observed in 40 nM of QDs, although it was not statistically significant. Interestingly, peroxidase activity was more prone to influence by QDs, as there was an over 60% decrease in its activity upon exposure of the bacteria to 40 nM of QDs. A decrease in SOD and peroxidase activity was accompanied by a decrease in survival of the bacteria and an increase in lipid peroxidation (thiobarbituric acid reactive species assay) and protein carbonyl—evidence of protein oxidation [58]. More information on the influence of SOD on CdTe QDs was obtained from thorough research by Sun et al., who aimed to describe the molecular mechanism of the change in Cu,Zn-SOD activity as a result of exposure to N-acetyl-L-cysteine-capped CdTe QDs (QDs-612) in vitro. Relative Cu,Zn-SOD activity decreased to 89.6% after the incubation of 50 µM of porcine erythrocyte Cu,Zn-SOD with 0.08 µM of QDs, and continued to decrease upon an increase in QDs concentration to 0.2 µM (79.5%). It was proved that QDs-612 affect the amino acid residues of the main polypeptide, breaking the hydrogen bonding, thus unfolding the structure of the enzyme [35]. Singh et al. [59] carried out research on prostate cancer cells, in vivo, assaying for SOD activity among many other markers of oxidative stress. In this case, the cells were exposed to biosurfactant-stabilized CdS QDs (bsCdS). The change in SOD activity due to exposure to QDs was referred to the change in SOD activity due to exposure to Cd(NO_3_)_2_. In this research, an increase in SOD activity was observed after incubating cells with QDs for 24 h or 48 h. The lowest concentration of QDs needed to obtain this effect was 10 µg/mL [59]. Interestingly, the cadmium salt proved to increase SOD activity at a lower rate—the increase in SOD activity after incubation with 25 µM of Cd(NO_3_)_2_ was statistically significant, although lower, compared with the increase observed after incubation with 10 µg/mL of QDs. Likewise, our results show the activation of SOD by QDs, although, in our research, the type of QD was different (CdTe QDs vs. CdS QDs).

The ability of QDs to emit light (fluorescence) makes it possible to label proteins or DNA. Effective and easy labeling of proteins with QDs offers greater opportunities for research on the importance of proteins in the course of diseases.

The assessment of the labeling potential of SOD1 with the use of CdTe QDs was carried out by analyzing the complexes using CE with LIF detection. Based on the conducted electrophoretic separations, the intensity of QDs-SOD1 fluorescence is influenced by the concentration of SOD1. In the case of SOD1–G-QD complexes, the strength of the fluorescence signal increased with decreasing SOD1 concentration. In contrast, the signal strength is similar for Y-QDs complexes with the four SOD1 dilutions. A similar relationship regarding the influence of the biomolecule concentration on the intensity of fluorescence was described by Stanisavljevic et al. [60]. The analysis concerned the interaction of chicken genomic DNA with CdTe QDs using CE and the studies showed that DNA concentration has an effect on the height of the fluorescence peak.

The migration time of free QDs and their complexes with SOD1 varied depending on the type of the used QDs. In the case of complexes of G-QDs with SOD1, the migration time differed slightly. Complexes of Y-QDs with SOD1, whose peaks are shifted to the left compared with the Y-QDs peak, were characterized by a shorter migration time. In turn, Tang et al. [61] analyzed the complex of QDs with DNA aptamers using CE. These analyses indicate that the type of the used buffer, its concentration, its pH, and the internal diameter of the capillary affect the separation time and the value of fluorescence intensity. The importance of pH in CE was also described by Huang et al. [62]. They investigated the relationship of the migration of complexes of QDs with albumin from bovine serum. As the buffer’s pH increased, the difference between the migration time of the complex of QDs with albumin and the QDs themselves increased. This is due to the negative electric charges of the formed complexes, which at a higher pH cause faster migration of the complexes compared with QDs alone [62]. Matczuk et al. [63] described the optimization of CE conditions to study the interaction of QDs with albumin. The complexation of QDs with albumin, which significantly increased the particle size, caused a decrease in its mobility. Free QDs that move towards the anode appear on the electrophoregram much faster (at 9.1 min) than the QD–albumin complex (at 11.2 min). The difference in the migration time of free QDs versus QDs–Tf (QDs–transferrin complex) was described by Guan et al. [64]. In their analysis, QDs complexes were characterized by a shorter migration time compared with the free QDs. The electrophoretic mobility of negatively charged QDs resulted in migration towards the anode. The separation of sample components is influenced by the sum of the vectors of two phenomena: electrophoresis and electroosmosis. Due to the electroosmotic flow, all ions during the migration moved towards the cathode along the capillary, passing through the detector window, where the counted signals were recorded in the form of the detector signal over time. Therefore, QDs with lower electrophoretic mobility showed a shorter migration time due to the electroosmotic mobility of the entire electrolyte mass. QDs–Tf complexes had a lower negative surface charge than QDs alone, and thus lower electrophoretic mobility. Hence, their migration time was shorter [64]. In contrast, Janu et al. [65] analyzed QDs IgY and QDs IgG conjugates using CE with both LIF detection and a UV detector. QDs IgY complexes showed the same electromigration properties as the unconjugated QDs. In contrast, the migration time of QDs IgG complexes was longer than that of QDs alone. After the binding of QDs to IgG, the migration time was extended from 4.1 min to 5 min.

## 4. Materials and Methods

### 4.1. Materials

Mercaptosuccinic acid, cadmium acetate dehydrate, sodium tellurite, and sodium borohydride were purchased from Sigma Aldrich. CdTe QDs were synthesized, using the microwave irradiation reduction method, as described elsewhere [66]. Cadmium chloride was obtained from Fluka (Darmstadt, Germany) (cat. no. 20899). Lyophilized bovine SOD1 was obtained from Sigma Aldrich (Darmstadt, Germany) (cat. no. S9697-15KU). Sodium carbonate, sodium bicarbonate, and EDTA were obtained from: POCH (cat. no. 1331-11-810360-2), Sigma-Aldrich (cat. no. S 6014-1KG), and Serva (Heidelberg, Germany) (cat. no. 11282), respectively. Hydrochloric acid and L-epinephrine were obtained from ChemPur (Piekary Śląskie, Poland) (cat. no. WE 231-595-7) and Serva (Heidelberg, Germany) (cat. no. 10980), respectively.

### 4.2. Methods

#### 4.2.1. Synthesis of CdTe QDs and SOD–QDs Complexes Preparation

Cadmium acetate dehydrate (0.044 g) was dissolved in 76 mL of MiliQ water in a 200 mL beaker on a magnetic stirrer. To this solution, 1 mL of the aqueous solution of mercaptosuccinic acid (60 mg/mL) was added, followed by 1.8 mL of 1 M ammonium solution. Then, the aqueous solution of sodium tellurite (5.5 mg) was added. Finally, after several minutes, 40 mg of sodium borohydride was added to the solution. The final solution was stirred for 1 h, its volume was adjusted to 100 mL with the addition of water, and it was divided into samples of 2 mL. Each sample was irradiated in a Multiwave 300 microwave oven (Anton Paar GmbH, Graz, Austria) for 10 min (300 W). According to this preparation procedure, CdTe QDs with the emission colors green, yellow, and red were obtained, depending on the temperature used: 70 °C (green), 90 °C (yellow), and 130/120°C (red), as described elsewhere [54].

The SOD1–QDs complexes of green and yellow QDs with four different SOD1 concentrations were prepared. The concentrations of SOD1 were as follows: 1.2 µg/mL, 4 µg/mL, 40 µg/mL, and 400 µg/mL.

#### 4.2.2. Dynamic Light Scattering

CdTe QDs were characterized in terms of average particle hydrodynamic perimeter and Zeta potential. The first parameter was determined with the use of the dynamic light scattering method (Zetasizer Nano ZS, Malvern, UK) at 25 °C. Samples with QDs were pre-diluted 1:100 with deionized water. Bovine superoxide dismutase was prepared by dissolving 7.2 mg of SOD in 720 µL of distilled water. The solution was then diluted 8000-fold to obtain a concentration of 1.25 µg/mL. The refractive index set up for these measurements was 1.59. The Zeta potential was also measured using Zetasizer Nano ZS. 

#### 4.2.3. Fluorescence Spectra Measurement

Fluorescence spectra of each type of QD were measured with the use of a Varioskan Lux Multimode Microplate Reader (Thermo Scientific, Waltham, MA USA) using SkanIt Software 4.1 for Microplate Readers RE, ver. 4.1.00043. The measurements were carried out in a NUNC Optical 96 Plate under the following conditions: 200 µL of the sample, an automatic 100 s measurement time, and an automatic dynamic range, starting from 400 nm to 850 nm, analyzed from the top wavelength.

#### 4.2.4. Assaying for SOD1 Activity

SOD1 activity was assayed with the use of the epinephrine method [67]. The assay was held at 30 °C, in 50 mM carbonate buffer, with 100 µM of EDTA. L-epinephrine (10 mM) was dissolved in 10 mM hydrochloric acid. In this method, epinephrine, upon exposition to alkaline pH (10.2), is autooxidized to adrenochrome, yielding superoxide (side product), which is scavenged by SOD [67]. The quantity of the product was determined by absorption spectroscopy (λ = 480 nm) using a Specord 40 spectrophotometer (Analytik Jena, Jena, Germany). The highest rate of adrenochrome formation was estimated. In the presence of SOD, the rate of epinephrine autooxidation is decreased due to the ongoing dismutation process. One unit of SOD activity is equal to a 50% decrease in the rate of adrenochrome formation. The influence of CdCl_2_ or CdTe QDs on SOD1 was determined using the aforementioned assay. Interference with the assay was taken into account when summarizing the data by subtracting the % of the change in rate when only QDs were present in the reaction mixture (SOD1 was absent) from the change in rate when both QDs and Cu,Zn-SOD, after pre-incubation, were present in the reaction mixture. The concentration of SOD1 in the reaction solution was 40 ng/mL. Before each assay, SOD1 was pre-incubated with aqueous solutions of CdCl_2_ or CdTe QDs, for 30 min, at 30 °C. Then, the pre-incubated solution was added to the reaction mixture. All electrophoretic separations were performed three times.

#### 4.2.5. Capillary Electrophoresis 

Electrophoretic separations were performed on a Beckman Coulter capillary electrophoresis PA 800 plus Pharmaceutical Analysis System with UV (200 nm) and LIF detection (λex = 488 nm, λem = 600 nm) using a 600 ± 40 nm emission band-pass filter (Edmund Optics, Barrington, NJ, USA65–736). An uncoated fused silica capillary with a total length of 60 cm and an internal diameter of 75 μm (Beckman Coulter, Fullerton, CA, USA, cat. no. 338454) was used.

SOD1 separation was performed using CE with a UV detector at 200 nm. After the capillary installation and calibration of the detector, the capillary was conditioned by rinsing first with NTMP separation buffer pH = 7.2 (30 s at 50 psi and 1 min at 50 psi). The course of electrophoretic separation was as follows: rinsing with 0.1 M NaOH (pressure 20 psi, 1 min) and with separation buffer (20 psi, 2 min). The hydrodynamic injection of the sample lasted for 8 s at 0.5 psi, with protective water post-injection (0.1 psi, 10 s). The separation process of the SOD1 sample was carried out at a voltage of 20 kV for 19.5 min, then the capillary was rinsed with separation buffer for 0.5 min (50 psi). 

The QDs and SOD–QDs complexes separation study was performed using CE with an LIF detector. After the capillary installation and calibration of the LIF detector, the capillary was conditioned by rinsing first with 0.1 M NaOH (1 min at 20 psi), then conditioned with a separation buffer (20 mM Na_2_B_4_O_7_∗10H_2_O, pH = 9.2) (for 2 min at 20 psi). The hydrodynamic injection of the sample lasted for 10 s at 0.5 psi, with protective water post-injection (0.1 psi, 10 s). The separation process of the sample was carried out at a voltage of 20 kV for 25 min, then the capillary was rinsed with separation buffer for 0.5 min (20 psi).

After each separation, the shutdown conditions were applied by rinsing with 0.1 M NaOH (20 psi, 2 min) and with water (20 psi, 2 min). All electrophoretic separations were performed three times. Karat 32 ver. 9.0 software (Beckman Coulter Inc., Fullerton, CA, USA) was used to acquire and analyze all data.

#### 4.2.6. Statistical Analysis

Statistical calculations were done using the Statistica Software Package, version 13.0 (Polish version: StatSoft, Krakow, Poland). The normality of the distributions of data was assessed by the Shapiro–Wilk W test. To test the differences between the two groups, the parametric Student’s t-test (normal distribution) or nonparametric U Mann–Whitney test for continuous variables was used. The homogeneity of variance was tested with Levene’s test. The differences between the examined groups were assessed using a one-way Analysis of Variance (ANOVA) with Duncan’s multiple comparison test (normal distribution) or the Kruskal–Wallis test (lack of a normal distribution). In all instances, *p* < 0.05 was considered statistically significant.

## 5. Conclusions

QDs may activate Cu,Zn-SOD, which would mean that QDs could also have an indirect antioxidant effect in vivo. Moreover, this activation may depend on the size and potential of QDs, which could mean that in the future we might be able to selectively alter the activity of specific enzymes by choosing QDs with specific characteristics. To confirm the cause of this phenomenon, more research is needed. In addition, due to their properties, QDs can be used to label SOD, whose concentration and activity have an impact on the occurrence of certain diseases. This was confirmed by the differences in the values of the fluorescence intensity of QDs and their complexes with different concentrations of SOD1 in the study using CE with LIF detection. The interaction between QDs and SOD was demonstrated, but further analysis is needed to confirm it and determine what type of interaction it is. This will offer the opportunity to follow the influence of SOD on the mechanisms of some diseases as well as to confirm the importance of the use of QDs in medicine and its related applications.

## Data Availability

The data presented in this study are available on request from the corresponding author.

## References

[1] Chan W.C., Maxwell D.J., Gao X., Bailey R.E., Han M., Nie S. (2002). Luminescent quantum dots for multiplexed biological detection and imaging. Curr. Opin. Biotechnol..

[2] Nirmal M., Brus L. (1999). Luminescence photophysics in semiconductor nanocrystals. Acc. Chem. Res..

[3] Alivisatos A.P. (1996). Semiconductor clusters, nanocrystals, and quantum dots. Science.

[4] Olkhovets A., Hsu R.-C., Lipovskii A., Wise F.W. (1998). Size-dependent temperature variation of the energy gap in lead-salt quantum dots. Phys. Rev. Lett..

[5] Leatherdale C.A., Woo W.-K., Mikulec F.V., Bawendi M.G. (2002). On the absorption cross section of cdse nanocrystal quantum dots. J. Phys. Chem. B..

[6] Michalet X., Pinaud F.F., Bentolila L.A., Tsay J.M., Doose J., Li J.J., Sundaresan G., Wu A.M., Gambhir S.S., Weiss S. (2005). Quantum dots for live cells, in vivo imaging, and diagnostics. Science.

[7] Wang G., Li Z., Ma N. (2018). Next-generation DNA-functionalized quantum dots as biological sensors. ACS Chem. Biol..

[8] Inoshita T., Sakaki H. (1997). Density of states and phonon-induced relaxation of electrons in semiconductor quantum dots. Phys. Rev. B..

[9] Hoshino A., Fujioka K., Oku T., Suga M., Sasaki Y.F., Ohta T., Yasuhara M., Suzuki K., Yamamoto K. (2004). Physicochemical properties and cellular toxicity of nanocrystal quantum dots depend on their surface modification. Nano Lett..

[10] Mashinchian O., Johari-Ahar M., Ghaemi B., Rashidi M., Barar J., Omidi Y. (2014). Impacts of quantum dots in molecular detection and bioimaging of cancer. Bioimpacts.

[11] Sun C., Cao Z., Wu M., Lu C. (2014). Intracellular tracking of single native molecules with electroporation-delivered quantum dots. Anal. Chem..

[12] Lisse D., Richter C.P., Drees C., Birkholz O., You C., Rampazzo E., Piehler J. (2014). Monofunctional stealth nanoparticle for unbiased single molecule tracking inside living cells. Nano Lett..

[13] Komatsuzaki A., Ohyanagi T., Tsukasaki Y., Miyanaga Y., Ueda M., Jin T. (2015). Compact halo-ligand-conjugated quantum dots for multicolored single-molecule imaging of overcrowding GPCR proteins on cell membranes. Small.

[14] Varela J.A., Dupuis J.P., Etchepare L., Espana A., Cognet L., Groc L. (2016). Targeting neurotransmitter receptors with nanoparticles in vivo allows single-molecule tracking in acute brain slices. Nat. Commun..

[15] Resch-Genger U., Grabolle M., Cavaliere-Jaricot S., Nitschke R., Nann T. (2008). Quantum dots versus organic dyes as fluorescent labels. Nat. Methods.

[16] Stroh M., Zimmer J.P., Duda D.G., Levchenko T.S., Cohen K.S., Brown E.B., Scadden D.T., Torchilin V.P., Bawendi M.G., Fukumura D. (2005). Quantum dots spectrally distinguish multiple species within the tumor milieu in vivo. Nat. Med..

[17] Gao X., Cui Y., Levenson R.M., Chung L.W., Nie S. (2004). In vivo cancer targeting and imaging with semiconductor quantum dots. Nat. Biotechnol..

[18] Levene M.J., Dombeck D.A., Kasischke K.A., Molloy R.P., Webb W.W. (2004). In vivo multiphoton microscopy of deep brain tissue. J. Neurophysiol..

[19] Akerman M.E., Chan W.C., Laakkonen P., Bhatia S.N., Ruoslahti E. (2002). Nanocrystal targeting in vivo. Proc. Natl. Acad. Sci. USA.

[20] Voura E.B., Jaiswal J.K., Mattoussi H., Simon S.M. (2004). Tracking metastatic tumor cell extravasation with quantum dot nanocrystals and fluorescence emission-scanning microscopy. Nat. Med..

[21] Drbohlavova J., Adam V., Kizek R., Hubalek J. (2009). Quantum dots—Characterization, preparation and usage in biological systems. Int. J. Mol. Sci..

[22] Stanisavljevic M., Krizkova S., Vaculovicova M., Kizek R., Adam V. (2015). Quantum dots-fluorescence resonance energy transfer-based nanosensors and their application. Biosens. Bioelectron..

[23] Manabe N., Hoshino A., Liang Y., Goto T., Kato N., Yamamoto K. (2006). Quantum dot as a drug tracer in vivo. IEEE Trans. NanoBiosci..

[24] Bagalkot V., Zhang L., Levy-Nissenbaum E., Jon S., Kantoff P.W., Langer O.C. (2007). Quantum dot−aptamer conjugates for synchronous cancer imaging, therapy, and sensing of drug delivery based on bi-fluorescence resonance energy transfer. Nano Lett..

[25] Zrazhevskiy P., Sena M., Gao X. (2010). Designing multifunctional quantum dots for bioimaging, detection, and drug delivery. Chem. Soc. Rev..

[26] Probst C.E., Zrazhevskiy P., Bagalkot V., Gao X. (2013). Quantum dots as a platform for nanoparticle drug delivery vehicle design. Adv. Drug Deliv. Rev..

[27] Zhang L., Shang L., Dong S. (2008). Sensitive and selective determination of Cu2+ by electrochemiluminescence of CdTe quantum dots. Electrochem. Commun..

[28] Chen S., Zhang X., Zhang Q., Hou X., Zhou Q., Yan J., Tan W. (2011). CdSe quantum dots decorated by mercaptosuccinic acid as fluorescence probe for Cu2+. J. Lumin..

[29] Dutta P., Saikia D., Adhikary N.C., Sarma N.S. (2015). Macromolecular systems with MSA-capped CdTe and CdTe/ZnS core/shell quantum dots as superselective and ultrasensitive optical sensors for picric acid Explosive. ACS Appl. Mater. Interfaces.

[30] Yuan J., Guo W., Yin J., Wang E. (2009). Glutathione-capped CdTe quantum dots for the sensitive detection of glucose. Talanta.

[31] Yuan J., Guo W., Wang E. (2008). Utilizing a CdTe quantum dots−enzyme hybrid system for the determination of both phenolic compounds and hydrogen peroxide. Anal. Chem..

[32] Wang X., Sun X., Lao J., He H., Cheng T., Wang M., Wang S., Huang F. (2014). Multifunctional graphene quantum dots for simultaneous targeted cellular imaging and drug delivery. Colloids Surf. B Biointerfaces.

[33] Zhao M.-X., Zhu B.-J. (2016). The research and applications of quantum dots as nano-carriers for targeted drug delivery and cancer therapy. Nanoscale Res. Lett..

[34] Lewandowski Ł., Kepinska M., Milnerowicz H. (2018). Inhibition of copper-zinc superoxide dismutase activity by selected environmental xenobiotics. Environ. Toxicol. Pharmacol..

[35] Sun H., Cui E., Liu R. (2015). Molecular mechanism of copper-zinc superoxide dismutase activity change exposed to N-acetyl-L-cysteine-capped CdTe quantum dots-induced oxidative damage in mouse primary hepatocytes and nephrocytes. Environ. Sci. Pollut. Res..

[36] McCord J.M., Fridovich I. (1969). Superoxide Dismutase. An enzymic function for erythrocuprein (hemocuprein). J. Biol. Chem..

[37] Fukai T., Ushio-Fukai M. (2011). Superoxide dismutases: Role in redox signaling, vascular function, and diseases. Antioxid. Redox. Signal..

[38] Lewandowski Ł., Kepinska M., Milnerowicz H. (2019). The copper-zinc superoxide dismutase activity in selected diseases. Eur. J. Clin. Invest..

[39] Younus H. (2018). Therapeutic potentials of superoxide dismutase. Int. J. Health Sci..

[40] Samia A.C., Chen X., Burda C. (2003). Semiconductor Quantum Dots for Photodynamic Therapy. J. Am. Chem. Soc..

[41] Ipe B.I., Lehnig M., Niemeyer C.M. (2005). On the generation of free radical species from quantum dots. Small.

[42] Lai L., Lin C., Xu Z.-Q., Han X.-L., Tian F.-F., Mei P., Li D.-W., Ge Y.-S., Jiang F.-L., Zhang Y.-Z. (2012). Spectroscopic studies on the interactions between CdTe quantum dots coated with different ligands and human serum albumin. Spectrochim. Acta Part. A Mol. Biomol. Spectrosc..

[43] Huang S., Qiu H., Lu S., Zhu F., Xiao Q. (2015). Study on the molecular interaction of graphene quantum dots with human serum albumin: Combined spectroscopic and electrochemical approaches. J. Hazard. Mater..

[44] Shukla G.S., Hussain T., Srivastava R.S., Chandra S.V. (1989). Glutathione peroxidase and catalase in liver, kidney, testis and brain regions of rats following cadmium exposure and subsequent withdrawal. Ind. Health.

[45] Roméo M., Bennani N., Gnassia-Barelli M., Lafaurie M., Girard J.P. (2000). Cadmium and copper display different responses towards oxidative stress in the kidney of the sea bass Dicentrarchus labrax. Aquat. Toxicol..

[46] Pruell R.J., Engelhardt F.R. (1980). Liver cadmium uptake, catalase inhibition and cadmium thionein production in the killifish (Fundulus Heteroclitus) induced by experimental cadmium exposure. Mar. Environ. Res..

[47] Jamall I.S., Crispin Smith J. (1985). Effects of cadmium on glutathione peroxidase, superoxide dismutase, and lipid peroxidation in the rat heart: A possible mechanism of cadmium cardiotoxicity. Toxicol. Appl. Pharmacol..

[48] Huang Y.-H., Shih C.-M., Huang C.-J., Lin C.-M., Chou C.-M., Tsai M.-L., Liu T.P., Chiu J.-F., Chen C.-T. (2006). Effects of cadmium on structure and enzymatic activity of Cu,Zn-SOD and oxidative status in neural cells. J. Cell Biochem..

[49] Zhu J.Y., Chan K.M. (2012). Mechanism of cadmium-induced cytotoxicity on the ZFL zebrafish liver cell line. Metallomics.

[50] Banni M., Chouchene L., Said K., Kerkeni A., Messaoudi I. (2011). Mechanisms underlying the protective effect of zinc and selenium against cadmium-induced oxidative stress in zebrafish Danio rerio. Biometals.

[51] Nyyssönen K., Porkkala-Sarataho E., Kaikkonen J., Salonen J.T. (1997). Ascorbate and urate are the strongest determinants of plasma antioxidative capacity and serum lipid resistance to oxidation in Finnish men. Atherosclerosis.

[52] Wang G., Su X., Yang S., Jia Y., Li D. (2012). The double-effect mechanism between Fe_3_O_4_ nanoparticles and MSA-capped CdTe QDs. J. Lumin..

[53] Yu X., Liu J., Zuo S., Yu Y., Cai K., Yang R. (2013). Application of mercaptosuccinic acid capped CdTe quantum dots for latent fingermark development. Forensic Sci. Int..

[54] Guszpit E., Krizkova S., Kepinska M., Rodrigo M.A., Milnerowicz H., Kopel P., Kizek R. (2015). Fluorescence-tagged metallothionein with CdTe quantum dots analyzed by the chip-CE technique. J. Nanopart. Res..

[55] Wang J., Zhang H., Zhang T., Zhang R., Liu R., Chen Y. (2015). Molecular mechanism on cadmium-induced activity changes of catalase and superoxide dismutase. Int. J. Biol. Macromol..

[56] Zeng G.-M., Chen A.-W., Chen G.-Q., Hu X.-J., Guan S., Shang C., Lu L.-H., Zou Z.-J. (2012). Responses ofp chrysosporium to toxic pollutants: Physiological flux, oxidative stress, and detoxification. Environ. Sci. Technol..

[57] Derfus A.M., Chan W.C., Bhatia S.N. (2004). Probing the cytotoxicity of semiconductor quantum dots. Nano Lett..

[58] Lu Z., Li C.M., Bao H., Qiao Y., Toh Y., Yang X. (2008). Mechanism of antimicrobial activity of CdTe quantum dots. Langmuir.

[59] Singh B.R., Singh B.N., Khan W., Singh H.B., Naqvi A.H. (2012). ROS-mediated apoptotic cell death in prostate cancer LNCaP cells induced by biosurfactant stabilized CdS quantum dots. Biomaterials.

[60] Stanisavjevic M., Chomoucka J., Dostalova S., Krizkova S., Vaculovicova M., Adam V., Rene K. (2014). Interactions between CdTe quantum dots and DNA revealed by capillary electrophoresis with laser-induced fluorescence detection. Electrophoresis.

[61] Tang T., Deng J., Zhang M., Shi G., Zhou T. (2016). Quantum dot- DNA aptamer conjugates coupled with capillary electrophoresis: A universal strategy for radiometric detection of organosphorus pesticides. Talanta.

[62] Huang X., Weng J., Sang F., Song X., Cao C., Ren J. (2006). Characterization of quantum dot bioconjugates by capillary electrophoresis with laser-induced fluorescent detection. J. Chromatogr. A.

[63] Matczuk M., Legat J., Timerbaev A.R., Jarosz M. (2016). A sensitive and versatile method for characterization of protein- mediated transformations of quantum dots. Analyst.

[64] Guan L.-Y., Li Y.-Q., Lin S., Zhang M.-Z., Chen J., Ma Z.-Y., Zhao Y.-D. (2012). Characterization of CdTe/CdSe quantum dots- transferrin fluorescent probes for cellular labeling. Anal. Chim. Acta.

[65] Janu L., Stanisavljevic M., Krizkova S., Sobrova P., Vaculovicova M., Kizek R., Adam V. (2013). Electrophoretic study of peptide-mediated quantum dot-human immunoglobulin bioconjugation. Electrophoresis.

[66] Duan J., Song L., Zhan J. (2009). One-pot synthesis of highly luminescent CdTe quantum dots by microwave irradiation reduction and their Hg 2+ -sensitive properties. Nano Res..

[67] Misra H., Fridovich I. (1972). The role of superoxide anion in the autooxidation of epinephrine and a simple assay for superoxide dismutase. J. Biol. Chem..

